# Hazard Ratio and Hazard Index as Preliminary Estimators Associated to the Presence of Furans and Alkylfurans in Belgian Foodstuffs

**DOI:** 10.3390/foods11162453

**Published:** 2022-08-14

**Authors:** Zouheir Alsafra, Georges Scholl, Bruno De Meulenaer, Gauthier Eppe, Claude Saegerman

**Affiliations:** 1 Mass Spectrometry Laboratory, MolSys Research Unit, University of Liege, Allée de la Chimie 3, B-6c Sart-Tilman, B-4000 Liege, Belgium; 2 Department of Food Safety and Food Quality, Nutrifoodchem Unit, Faculty of Bioscience Engineering, Ghent University, Coupure Links 653, B-9000 Ghent, Belgium; 3 Research Unit in Epidemiology and Risk Analysis Applied to Veterinary Sciences (UREAR), Fundamental and Applied Research for Animal Health (FARAH) Centre, Faculty of Veterinary Medicine, University of Liege, Quartier Vallée 2, Avenue de Cureghem 7A, B-42, Sart-Tilman, B-4000 Liege, Belgium

**Keywords:** furan, alkyl furans, contamination levels, co-occurrence pattern, hazard ratio, hazard index, Belgium, food chain

## Abstract

This paper provides an estimation of the hazard related to the presence of furan and five alkyl furans (2- and 3-methylfuran, 2-ethylfuran, 2,5- and 2,3-dimethylfuran) in foodstuffs available in the Belgian market. To achieve this objective, a specific sampling plan was designed to ensure that the samples collected (n = 1003) represent the diversity of the Belgian food chain. Herein, the concepts of the Hazard Ratio of a sample (HRs) and the Hazard Index of a sample (HIs) were introduced to primarily characterize the hazard related to the co-occurrence of these compounds. The HRs was measured as the ratio of the potential daily exposure to a substance (expressed in mg/Kg of food) to both the 10% reference dose level for chronic effects (expressed in mg/(kg b.w*day)) and the human standard weight (expressed in kg). Whereas the HIs is the sum of the HRs of compounds that affect the same target organ/system, a hazard index greater than one indicates a highly contaminated matrix that could induce a hazard. It is an alarm indicating that additional attention should be given to this matrix. This may involve additional analyses to confirm the high level, to identify sources, etc. It is also an alarm for the risk assessor to be very careful with flagged matrices and to avoid combination with other matrices. The HIs highlight a relatively low concern for all foods analyzed (HI median < 1.0) with a relatively higher suspected hazard for coffee drinks (HI median = 0.068, HI max = 0.57). This preliminary estimation of the potential hazard suggests that coffee beverages should be examined in more detail in a full risk assessment and that coffee consumption should be taken with caution given the levels of furan and alkylfurans reported in this study.

## 1. Introduction

Furan (C_4_H_4_O) and its methyl derivatives are known to be present in a wide range of foods especially in those undergoing heat treatment [1,2]. These compounds exhibit some harmful effects, and the parent furan itself is classified by the IARC as a possible human carcinogen (group 2B) [3,4]. Since the report published by the US Food and Drug Administration on the occurrence of furan in foods [5], it has become an increasing matter of concern and thus different aspects related to this compound have been studied. These aspects include mainly the toxicity [6,7,8], the formation pathways [9,10,11], the occurrence in foods [12,13,14], the risk assessment [15,16,17,18], and the analytics [19,20]. However, these data mainly refer to furan, while limited data are available on its alkyl derivatives. Hence, the European commission asked Member States to provide more information on the contamination levels of these compounds in food [21,22].

Two main sampling strategies can be implemented to conduct a food survey on a food contaminant. The first one focuses on sampling specific food group(s) [23,24], while the second one is an exhaustive sampling of different foods groups [25]. The second one was used in the current work in order to give an overall estimate of food contamination with furan compounds to be used in a further work in the risk assessment.

A preliminary characterization of the hazard related to the co-occurrence of these compounds in food was introduced in this work as a starting point for further risk assessment.

The hazard characterisation was done firstly using the Hazard Ratio per sample (HRs) approach, which consists in comparing the intake determined in this work with the product of the available effective toxicological values and the standard body weight of human. Subsequently, the individual HRs of all furan compounds are combined into a global Hazard Index per sample analysed (HIs) [26,27,28].

In this work, two objectives are addressed. First, we aim to create a representative sampling plan for food samples collected in Belgium. Second, we aim to preliminary characterize the hazard associated with the co-occurrence of furan compounds (i.e., furan, 2-methylfuran (2-MeF), 3-methylfuran (3-MeF), 2-ethylfuran (2-EtF), 2,5-dimethylfuram (2,5-dMeF), and 2,3-dimethylfuran (2,3-dMeF)) in food.

This work is limited to preliminary hazard characterization, while a full risk assessment will be explored in detail in subsequent work based on EFSA approaches (EFSA, 2017).

## 2. Materials and Methods

### 2.1. Standard and Solutions Preparation

Furan was purchased from Sigma-Aldrich (St. Louis, MO, USA) with purity higher than 99%. 2-MeF, 3-MeF, 2-EtF, 2,5-dMeF, and 2,3-dMeF at purity higher than 99% were supplied by Toronto Research Chemical (Canada). Labelled standards including furan-d_4_, 2-MeF-d_6_ were provided from Campro Scientific (Germany); 3-MeF-d_3_ and 2,5-dMeF-d_3_ at purity higher than 99% were purchased from Toronto Research Chemical (Canada).

The term ‘furans’ used in this work refers to the sum of furan, 2-MeF, 3-MeF, 2-EtF, 2,5-dMeF, and 2,3-dMeF and is not related to the chlorinated dibenzofurans, which are often also referred to as “furans”.

To prepare working solutions, a 20 mL headspace (HS) vial is filled with methanol (Absolute, Biosolve, Dieuze, France), 10 µL of each analytical native standard are introduced via vial septa, then, 10 µL of this solution mix is introduced into another 20 mL HS vial filled with Milli-Q *^®^* water to reach a final concentration of about 200 ng/mL for each furan compound. All steps were carried out on an analytical balance. The first solution was prepared each 2 weeks and stored at −20 °C, while the second one was prepared every day and kept a +4 °C. The same procedure was applied for the deuterated analogues.

### 2.2. Samples Collection and Storage

To ensure the representativeness of the samples collected, the sampling procedures determined by the Commission Regulation (EC) No 333/2007 were followed [29]. For each item, three incremental samples (i.e., quantity of material taken from a single place in the lot or sub-lot) were collected from the major Belgian food retailers and precautions were taken during the collection of the samples to avoid any changes that could affect the contaminant levels. All information about the collection and the samples was recorded, such as the date of collection, location information, product category, brand and name of the product, nutritional composition, organic production method, packaging, and other visual information.

The samples were kept closed in the dark and at the temperature recommended by the manufacturer (i.e., −20 °C, 4 °C, or 25 °C) prior to analysis, to ensure that the contents were not altered. Sampling was carried out over two and a half years (March 2019–September 2021) to take into account the seasonal variation of the products.

### 2.3. Sample Preparation for the Analysis

Prior to analysis, samples were cooled to 4 °C to reduce the loss of volatile substances during sample opening. The samples were analyzed as purchased without any heat treatment. For sample intake, the incremental samples (n = 3) were homogenized or ground, and then, approximately 15 g from each of them were taken to make an aggregate sample. An appropriate amount of the aggregate sample and saturated brine solution (NaCl 26%) were sealed in a 20 mL HS vial. Typically, about 1 g was taken for most foods and 50–100 mg for ground coffee. Vials were spiked with internal standards mix through the septa of the caps and were vortexed for 1 min at 750 rpm before the extraction by HS-SPME.

### 2.4. Analytical Method

The analysis by headspace solid phase micro-extraction coupled to gas chromatography/mass spectrometry method (HS-SPME GC/MS) was performed according to [30] with slight modifications. In brief, for volatiles extraction, 50/30 µm of DVB/CAR/PDMS fibers (Supelco St. Louis, MO, USA) were used. The HS-SPME was performed by CTC Combi-Pal auto-sampler (CTC Analytics AG, Zwingen, Switzerland). A Peltier temperature controller was used to control the temperature of incubation and extraction of the samples. Desorption of the volatiles from the SPME fiber was done in the GC injection port (split/splitless) at 230 °C for 1 min. The cleaning of the fiber was done after each analysis in a conditioning chamber for 20 min at 280 °C.

The analysis was done using Trace-GC 2000 (Thermo-Scientific, Waltham, MA, USA) coupled to a PolarisQ ion-trap mass spectrometer (Thermo-Scientific, Waltham, MA, USA). The chromatographic separation was achieved using PoraBond-Q (25 m × 0.32 mm × 5 µm) column (Varian, Palo Alto, CA, USA) at a constant flow rate of 1.7 mL/min He (alpha gas 2, Air Liquid, Belgium). The GC temperature program begins at 45 °C during 1 min, ramp at 120 °C/min to 120 °C, hold for 20 min, followed by another temperature ramp of 10 °C/min until 260 °C and hold for 2 min resulting in a total run time equal to 37 min. The mass spectral acquisition was recorded in selected ion monitoring (SIM) mode.

To ensure the reliability of the results provided by this work, a validation plan was created prior to the analysis of the 1003 samples. In brief, this plan targeted mainly five food matrices mentioned in the EFSA report [17] including coffee, baby foods, chips and snacks, and cereal-based foods and beverages. The strategy of the validation was performed based on the European Commission decision n° 2002/657/EC [31] by evaluating the linearity of the calibration curves, the precision, the trueness, the specificity, and the limit of detection (LOD) and limit of quantification (LOQ). Then, the produced results were evaluated based on the criteria of the analytical performances recommended by the European commission for furan and methylfurans analysis [21]. The obtained LOQ for furan, 2-MeF, and 3-MeF is 1.8 µg/kg in coffee and 0.5 µg/kg in other food matrices. The LOQ of 2-EtF, 2,5-dMeF, and 2,3-dMeF is 4.5 µg/kg in coffee and 2.5 µg/kg in other foods. These values could slightly be changed depending on the nature of the analyzed food matrix.

The performances of the analytical method were checked by participating in international proficiency tests (PT) organized by the European Reference Laboratory for Process Contaminants. Three PT were performed to measure the demanded furan compounds in coffee, in baby food, and in cereals. Satisfactory results were obtained with z-score within the ±2 range. In addition, to ensure the daily performance of the method, in each injection sequence, one or two spiked samples with known amounts of native furans standards were added to ensure the recovery estimated by dividing the measured content to the fortification level.

The methods and analyses were performed under an ISO17025 system and the used method was accredited by BELAC (the Belgian accreditation body).

### 2.5. The Frequency of the Occurrence (Presence/Absence Pattern)

The frequency of occurrence of the furans was investigated to give a brief overview on their co-occurrence in the coffee versus other food groups without considering their concentrations in the food (considering presence when the concentration was above the LOQ of the sample).

The co-occurrence of furans was also further investigated using the Receiver Operator Characteristic (ROC) method. This method consists of evaluating the sensitivity and the proportion of false-positive (one minus specificity) of the model at the six possible cut-off (presence of 1 to 6 furans) (Figure S1).

### 2.6. Hazard Ratio and Hazard Index Calculation

The methodology used in this paper was based on the earlier work of Glorennec et al. (2001); Bonvallot et al. (2010) and Nedellec et al. (2012) [26,27,28].

The Hazard Ratio of a sample (HRs) is the ratio of the concentration of a specific chemical in a specific food item (i.e., sample, expressed in mg/kg) to the toxicological reference dose (TRD), the level at which no adverse effects are expected (expressed in mg/(kg_b.w._*day)). When the TRD is multiplied by the body weight (expressed in kg), the ratio becomes the fraction of the total amount of the chemical that 1 kg of product represents.

The TRD value used here is the benchmark dose (lower confidence limit) at 10% extra risk (BMDL_10_) to take into account the chronic effects. Several values of BMDL_10_ exist for furan based on the toxicity endpoint [17,32]; the value of 0.064 mg/(kg_b.w._*day) for non-neoplastic effects was used in this work as the most sensitive point of departure identified [33]. In addition, because there are no toxicological reference values reported, the same value was applied for other furan compounds targeted here considering similar chronic effects [17,32].

Regarding the body weight (BW), the value of 70 Kg was used as an estimation of the average BW of the European population [34]. Thus, the formula used for HRs is described as shown in the Equation (1).

Equation (1). The formula used in HRs calculation for each furan compound in one food sample.

(1) $$\mathrm{HRs}=\frac{\left[\mathrm{molecule}\right]\;\left(\frac{\mathrm{mg}}{\mathrm{kg}}\right)}{{\mathrm{BMDL}}_{10\;}*\;\mathrm{BW}}$$

For the Hazard Index *ratio* of a sample (HIs), it is the sum of hazard ratios for all targeted toxics in a specific food item that affect the same target organ or organ system (Equation (2)). As different toxins can cause similar adverse health effects, combining hazard quotients from different toxins is often appropriate and especially for a specific sample.

Equation (2). The formula used in HIs calculation for the six furans in one food sample.

(2) $$\mathrm{HIs}=\overset{i=6}{\underset{i=1}{\sum }}{\mathrm{HRs}}_{i}$$

Indeed, a HIs of less than 1.0 refers to a low contaminated sample, thus it is unlikely to result in adverse health effects. A HIs equal to or greater than 1.0, however, does not necessarily suggest a likelihood of adverse effects. The methodology was applied to prioritize efforts on specific food items of interest in a subsequent quantitative full risk exposure assessment.

### 2.7. Left Censoring

To deal with the contamination data lower than the Limit of Quantification (LOQ), different approaches could be used; the lower bound (LB), the middle bound (MB) or the upper bound (UB) [35]. In the LB, MB, and UB approaches, the results below the LOQ are replaced by zero, half-LOQ, and the LOQ itself, respectively. In this work, the MB approach was used assuming the hypothesis of a normal distribution of the results below the LOQ.

### 2.8. State of the Analysed Samples

For the occurrence data of furan compounds in the samples analyzed in this work, the concentrations were calculated in µg/kg of product as purchased following the strategy mentioned in the EFSA report [17]. Thus, for coffee and coffee imitates (n: 303), the occurrence data indicated in this work are mainly referred to solid samples (n: 280) and a limited number of results referred to beverages (n: 23).

Whereas, in the characterization of the hazard, the concentration of furan compounds in coffee referred to liquid state to compare the effects of the products as consumed. Therefore, a conversion step was performed prior to the characterization of the hazard by dividing the concentrations of furans in coffee (solid) by dilution and loss factors already determined by the EFSA [17].

### 2.9. Statistical Analysis

The comparison between the number of furans present in food groups was assessed using a Chi-squared test [36]. The co-occurrence of furans was investigated using Receiver Operator Characteristic (ROC) method [36].

The comparison of furans concentrations or HIs between food groups was assessed using a quantile median regression. The decimal logarithmic transformation was applied in some figures to be able to combine different concentrations scales. All analyses were performed using Stata SE 14.2 (Stata Corp, College Station, TX, USA) and JMP v10.0 (SAS, Cary, CA, USA), with a significance level of 0.05.

## 3. Results

This section describes the strategy used in this work to create the sampling plan to collect food samples, as well as the global contamination levels and co-occurrence of furans detected in the food analyzed in the survey, and an evaluation of the hazard associated to furans co-occurrence in different food groups.

### 3.1. Sampling Design

According to EFSA’s scientific opinion, there is a lack of information on alkyl furans levels in foods [17]. Therefore, a large-scale sampling plan was designed in this study (n: 1003 samples) to provide a comprehensive database for Belgium, to fulfil the European recommendations and to initiate a risk assessment. The sampling proposed here is based on three key parameters including previous contamination data, diversity, and appearance of the food.

First, the main food groups in the plan as well as the number of samples allocated for each group were proposed based on the level of furan contamination in foods already reported. According to the EFSA report, the group of people most exposed to furan is infants, mainly through consumption of ready-to-eat jarred or canned foods. Exposure of other population groups is mainly due to the consumption of coffee and grain-based foods, depending on age and consumer habits [17,37]. Therefore, special emphasis was paid in this work to these food groups by allocating a relatively high number of samples for each of them in the initial proposed sampling plan. Similarly, different main groups were proposed such as composite food, snacks, bakery products, etc.

Second, within each main group, several sub-groups were proposed according to the diversity of food products as shown in the Table 1. This parameter was evaluated by conducting a field investigation through the major food market chains. Nevertheless, due to COVID-19 constraints, those investigations were limited to coffee and baby foods sampling plans. For the remaining food groups, their sub-groups were proposed based on the number of different items present in the EFSA catalogue “FoodEx2” [38] as well as in the Food category system of the General Standard on Food Additives of Codex Alimentarius [39].

Third, for each food group, special attention was given to the dietary aspect such as organic, fair trade, and special diets. In 2019, Biowallonie published a report on the evolution of the organic market share in Wallonia (southern part of Belgium) [40]. It was only 1.5% in 2009, slowly increased to 3.2% in 2018, and reached 4.8% in 2019, which proves a growing attraction for organic food products. Therefore, as the weight of organic foodstuffs increased, about 10% of the samples were allocated to organic products.

The samples were collected randomly from the main food chains over a two-and-a-half year period, to avoid any one brand or food chain being tied to a province, but also to take into account for seasonal variations in products.

The proposed sampling plan was subsequently submitted to a Belgian committee of food safety experts, who reviewed it and came up with the final validated sampling plan that is summarised in Table 1.

### 3.2. Food Survey Results

The co-occurrence of the targeted furans was investigated in four steps. First, by evaluating the frequency of the presence in food. Second, by examining the volatile profile by looking at all possible correlations between furan concentrations. Third, by assessing the range of concentrations of each furan compound detected during the two and a half years of the survey. And finally, by evaluating the range of the total concentrations of furans in each food group.

#### 3.2.1. The Frequency of the Occurrence (Presence/Absence)

The frequency of the co-occurrence of furans detected in the coffee versus other food groups are displayed in Table S1. According to this table, the number of furan compounds is significantly higher in coffee than in other food groups (Chi-squared test (6 d.f.; α = 0.05) = 686; *p*-value < 0.001).

The percent of the presence/absence of individual compound is shown in Table S2. This table highlights that both furan and 2-MeF are the most abundant among the compounds targeted in this work where they are present in at least 80% of the analyzed samples. The next most abundant one is 3-MeF, followed by 2-EtF, 2,5-dMeF, and finally 2,3-dMeF, which was frequently <LOQ.

Concerning the ROC exploratory method, the cut-off (1) in the upper right corner of the ROC curve (Figure S1) indicates the presence of only one of the six compounds with a high sensitivity (100.0%), but also with a high false-positive (low specificity equal to 12.7%). Whereas the best cut-off is (5) at the upper left corner, with a high sensitivity (82.5%) and few false-positive (specificity of 96.9%) and where the majority of coffee patterns are distributed. This method highlights that the presence of five furans among the six furans targeted in this work can be used to discriminate coffee samples from other food samples. In addition, the area under curve is equal to 0.9386, meaning that 93.9% of coffee samples analyzed can be predicted using the current ROC method.

#### 3.2.2. The Volatile Profile of Furan Compounds in Specific Foods

The correlation between furan concentrations was investigated by evaluating the distribution of ratios between furan and its alkyls concentrations. The results obtained in this survey display that distinct profiles were observed in coffee and canned fish.

In coffee group, ratios were calculated for roasted coffee beans and ground coffee (n = 276). Whereas, coffee imitates, coffee derivatives (with additives such as milk or chocolate), and few samples of coffee beverages were not included in the ratio measurement as they increased the variability of the results. The medians of the concentrations of 2-MeF, 3-MeF, and 2.5-dMeF relative to the furan concentration were 3.71, 0.16, and 0.35, respectively, with a relative standard deviation (RSD%) of 19, 18, and 30% respectively. These values are consistent with those published by EFSA [17]. The distribution of these ratios was plotted in the Figure S2. The median of the ratio [2-EtF]/[furan] and [2,3-dMeF]/[furan] were lower than 0.03 with high variability (RSD > 30%), which is why the distributions were not provided.

In seafood products, relatively high levels of the 2-EtF were observed in canned fish sup-group (n = 29) as is detailed below. The median of the ratio 2-EtF/Furan and 2-EtF/2-MeF were 22.2 and 23.1 respectively (Figure S3).

#### 3.2.3. The Concentrations Range of Individual Compound

The concentrations of each compound were plotted in a box plot to give an overview of the concentrations range detected in the survey, regardless of the nature of the food. However, because of the difference in the concentration ranges detected in coffee and in other food matrices, they were plotted in two separated plots (Figure S4A,B). According to Figure S4A, the highest maximum level found for furan derivatives in coffee group is that for 2-MeF, followed by furan, and then 2,5-diMeF with concentration ranging from (LOQ-31547.5), (1.8–7404.5), and (LOQ-3200.2) µg/kg, respectively. These compounds were mainly present in coffee in its solid state.

In the other foods groups, the highest maximum level is for 2-EtF, followed by 2-MeF, and then furan with a concentration range situated between LOQ-1372.0, 293.0–and 919.9 µg/kg, respectively (Figure S4B).

#### 3.2.4. The Total Concentration of Furans Per Food Sample

**Figure 1:**
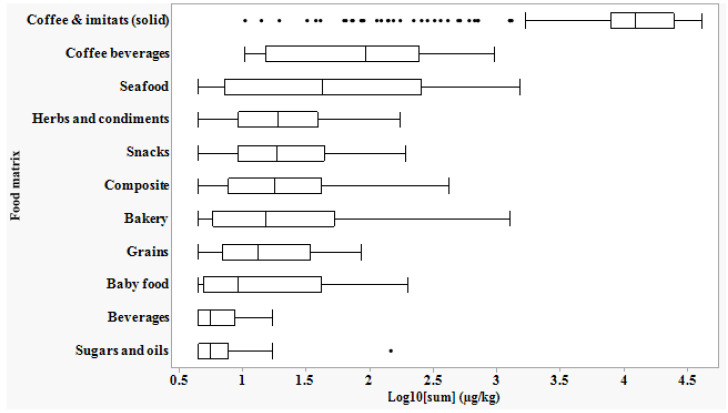
The Box plot of the logarithm of total concentration of furans detected in different food groups. Legend: The center line in the box plot represents the median; box limits represent the upper and lower quartiles; whiskers are 1.5× interquartile range; and points are outliers.

The concentrations of all compounds detected in each sample were combined and plotted against the food groups to display the distribution of the sum within the groups (Figure 1 and Table S3). The total amount of furans in coffee and coffee imitates (solid, n: 280) is significantly higher than that in other food groups (quantile median regression; *p*-value < 0.0001).

The total concentrations in this sup-group were evaluated in more detail (Figure S5).

### 3.3. The Hazard Characterization

**Figure 2:**
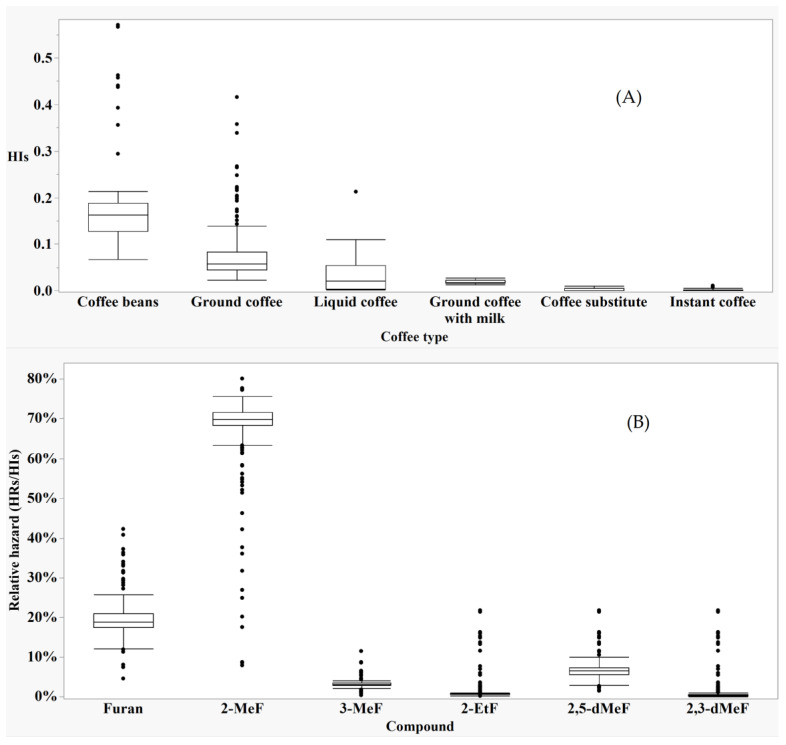
The distribution of the hazard indexes in coffee sup-groups (**A**), and the relative hazard distribution for furan compounds in coffee (**B**). Legend: The centre line in the Box plot represents the median; box limits represent the upper and the lower quartiles; whiskers are 1.5× interquartile range; and points are outliers.

The hazard index of a sample was calculated in different food matrices as described in the materials and methods section and summarised in Table S4. The medians of the HIs in all food groups were <1.0, with the highest median observed for coffee beverages (after applying the EFSA conversion factors on solid coffee). Therefore, the hazard associated with this group was further investigated. First, by evaluating the distribution of HIs within each coffee beverages sub-groups (Figure 2A), and then by evaluating the contribution of the HRs of the individual furan derivatives to the HIs in each coffee sample (Figure 2B).

Note that values of HIs indicated in Figure 2A are calculated for coffee beverages after applying suitable conversion factors to convert solid products (beans, ground, ground with milk, substitutes and instant) to liquids. While the HIs for liquid coffee sup-group (hot and cold conserved coffee) were calculated without conversion.

The HIs for furans in beverages prepared from beans or grounds, and liquid coffee (as purchased) were significantly different (*p*-value < 0.0001, using Wilcoxon multiple non-parametric comparisons). In contrast, there was no significant difference between liquid coffee and ground with milk, and between coffee substitute and instant coffee (Figure 2A).

As shown in (Figure 2B), taking the ratio between the HRs of individual compound to the HIs, 2-MeF and furan contribute most to the hazard evaluation, with significantly higher medians compared to other compounds present in coffee (quantile median regression; *p*-value < 0.0001).

## 4. Discussion

The results of the current paper suggest that furan and 2-MeF are the most abundant furans detected in the analyzed foods, while the less abundant one is 2.3-dMeF (Table S2). The difference in the amounts of furan compounds could be related to the mechanism of formation of these compounds as well as to the amounts of their precursors presents in food prior to processing [41].

In addition, according to the ROC curve, the presence of five furans among the six targeted molecules can be used as a predictor to discriminate coffee as a separate group. Based on the range of total furan concentrations in the food groups shown in Figure 1, it is clear that the sum is consistently below 200 µg/kg in the majority of food groups, except in coffee and some other groups.

In the coffee group (n = 303) and particularly in solid coffee, the total concentrations are significantly higher than in the other food groups (*p*-value < 0.0001) due to its pattern discussed above as well as to the high contribution of each compound, in particular 2-MeF (Figure S4A). Total concentrations of furan compounds in coffee beans are higher than in ground coffee (Figure S5), which could be explained by volatilization during industrial-scale grinding compared to beans that were ground prior to analysis. In contrast, total concentrations in the other coffee sub-groups are significantly lower due to lower furan compounds content resulting from the loss of volatiles during the process (freeze-drying in instant products), the dilution factor (e.g., powdered milk), or furan precursors such as in coffee substitutes. A significant difference was observed between the total amounts of furans in coffee (in its solid state) and coffee beverages (Figure 1). This difference is due to the evaporation during coffee preparation (e.g., brewing, percolation) and the dilution with water. Indeed, the consumer behaviour for coffee preparation has an important effect on the amount of furans retained in coffee beverage [42]. The way of coffee brewing for examples and the type of coffee machine (capsules, filters etc.) seem to influence significantly the final concentration in a cup of coffee [43].

In the other food groups, the total furans concentration was relatively high in some samples, especially in seafood, composites, and bakery products as described below. The high amount in seafood group (n = 53) can be explained by the contribution of 2-EtF (Figure S4B). The amount of this compound ranged from 50 to 600 µg/kg in canned and jarred fish such as sardine, mackerel, herring, and tuna, as well as tuna salad (n = 29); the highest level being in canned fish packed with tomato sauce (up to 1372 µg/kg) (Figure S4B). This is because raw fish products generally contain high amounts of polyunsaturated fatty acid, making them sensitive to oxidation during heat treatments [44]. The 2-EtF is thought to be derived from 2-hexenal, a typical oxidation of n-3 unsaturated fatty acids [45,46,47], which could explain its levels in processed fish. The low pH value also seems to induce 2-EtF formation as observed in canned fish with tomato sauce. This again can be linked to the reaction mechanism by which 2-EtF is formed from 2-hexenal, requiring a dehydration of a key intermediate, which is likely to be facilitated in acidic conditions [47]. The effect of acidic pH on furan has already been observed in fish sold in lemon juice [48].

This sum of furan concentrations was also relatively high in a few samples in the bakery group (n = 92), mainly in dark biscuits (n = 5), such as pretzels and Speculoos (i.e., cinnamon spiced caramelised biscuit), which could be related to the baking conditions as well as the Maillard reaction taking place during the process [49]. In these products, the 2-MeF contributes the most with a concentration range (270–900) µg/kg approximately (Figure S4B).

In the composite group (n = 107), the highest amount was found in canned meat stews and liquid soups, especially those containing meat balls (n = 6). In these products, the main contributor was 2-EtF followed by furan, which could again be explained by the effect of the sterilisation process on foods containing unsaturated fatty acids. In the baby food group, the higher contents were observed in snacks for infants followed with jarred baby food and cereals with medians of the total furan equal to 61.7, 47.7, and 10.3 µg/kg, respectively. The levels of furans in homemade food were always near to the LOQ and they were not included in Table S3.

Regarding the volatile profiles in coffee (Figure S2), the results suggest that coffee contains about 3.7 times more 2-MeF than furan, indicating that the main exposure to furan derivatives in coffee is due to 2-MeF. Compared to coffee, the main exposure to furans in canned fish is due to 2-EtF, which is frequently 22–23 times higher than furan and 2-MeF respectively (Figure S3). However, the level of 2-EtF in canned fish is at least two orders of magnitude lower than that of 2-MeF in coffee.

Based on the previous discussion, the main contributor to furanic compounds is coffee, due to its distinct pattern and the higher concentration of each compound.

However, relying only on the frequency of the presence in foods and the concentrations is not sufficient to express the hazard, but the toxicological values should be used to take into account the harmful effects of each compound [50]. According to the hazard index values presented in the Table S4, all food groups exhibit a low hazard (HIs median < 1.0) except for coffee and coffee imitates where the median is highest. The beverages prepared from coffee beans and ground coffee (with HIs median equal to 0.163 and 0.058 respectively) are the main hazard contributors (Figure 2A). In contrast, the hazard in the remaining coffee sub-groups is significantly lower due to the lower furan content resulting from the loss of volatiles during the process as described above in Figure S5.

On the other hand, as 2-MeF and furan are the most abundant furan compounds in coffee, they represent the main hazard contributors according to Figure 2B. considering similar undesirable effects.

Based on the discussion of the results presented in this work, the main potential hazard related to furan and alkyl furans in consumer foods are coffee, in particular coffee beans and ground coffee, due to the presence of 2-MeF and the parent furan.

## 5. Conclusions

A dedicated and specific sampling plan consisting of 1003 samples was described in this paper for the collection of samples in the Belgian food chains. The results of the food survey display that coffee is the foodstuff most contaminated with furan derivatives, but also the main food abundant with these compounds in terms of frequency of the occurrence. The total concentrations of these compounds in the other food groups are generally lower than 200 µg/kg except in canned fish, and some composite (canned meat and soup) and bakery products.

All food groups exhibit a low hazard (HIs median < 1.0) including coffee and coffee imitates, but with the highest median due to the high content and frequency of occurrence of furans in coffee. Further studies are needed to properly assess the risk of exposure to these compounds in food based on current consumption data.

## Figures and Tables

**Table 1:** A summary of the sampling plan (n: 1003).

Main Group	*n*	Sub-Groups
Coffee	303	Coffee beans, ground, instant (in capsules, pads or filters), coffee imitates, as well as coffee brew (hot and cold)
Baby food	135	Retail food, homemade, specific products (allergies)
Snacks	108	Chips, crisps, dough-based analogues, snacks other than chips, dairy snacks and confectionery
Composite food	107	Dishes including ready-to-eat meals, soups (instant and liquid), salads, spoonable desserts and ice creams, foods for particular diets (pharmaceutical products)
Bakery	92	Bread and similar, fine bakery wares, biscuit and crackers, particular bakery products (gluten free)
Grains and grains-based products	83	Cereals and cereal primary derivatives, breakfast cereals, pasta and similar products, particular products such as gluten free
Spices and condiments	67	Seasoning, savoury extracts and sauce ingredients, condiments
Seafood	53	Canned and cooked fish, seaweeds and other fish products
Beverages	41	Soft and energy drinks, alcoholic drinks (beers, sherry, liqueurs, and spirits), dairy products, tea and juice
Sugars and oils	14	Sugars and oils

## Data Availability

The data that support the findings of this study are available from the corresponding author upon request.

## Supplementary Materials

The following supporting information can be downloaded at: https://www.mdpi.com/article/10.3390/foods11162453/s1, Figure S1: The Receiver Operator Characteristic (ROC) curve for furans co-occurrence; Figure S2: The box plots and the histograms of the relative concentrations 2-MeF/furan (A), 3-MeF/furan (B) and 2,5-dMeF/furan (C) in coffee products (n = 276); Figure S3: The box plots and the histograms of the relative concentration 2-EtF/furan (A), 2-EtF/2-MeF (B) in processed fish products (n = 29); Figure S4: The Box plots of the Logarithm of the individual compound concentration in the coffee (A), and in other food groups (B); Figure S5: The box plots of the total concentrations of furan compounds in coffee products (solid) (n = 280); Table S1: The frequency of the number of furan compounds detected in coffee versus other foods; Table S2: The percent of the presence / absence of furan compounds (n: 1003); Table S3: The total concentration (µg/kg) of the selected furan compounds in the different food groups; Table S4: The hazard index for the selected furan compounds in the different food groups.
