# Supplementary material for: Hazard Ratio and Hazard Index as Preliminary Estimators Associated to the Presence of Furans and Alkylfurans in Belgian Foodstuffs
Source: Foods. 2022 Aug 14;11(16):2453. doi: 10.3390/foods11162453 (PMC9407134; doi:10.3390/foods11162453)
Supplement: Supplementary file 1 [file foods-11-02453-s001.zip › foods-1818496-supplementary.pdf]

foods

Supplementary Materials

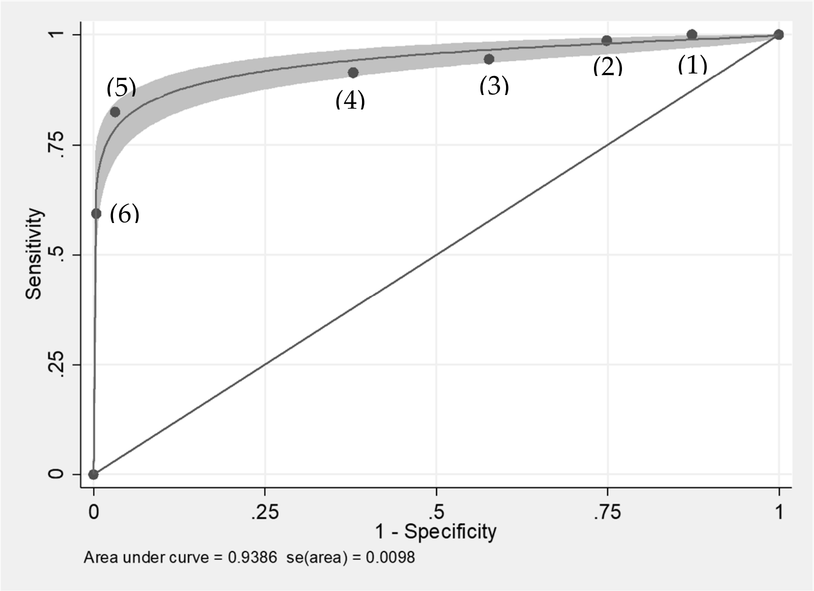

Figure S1: The Receiver Operator Characteristic (ROC) curve for furans co-occurrence

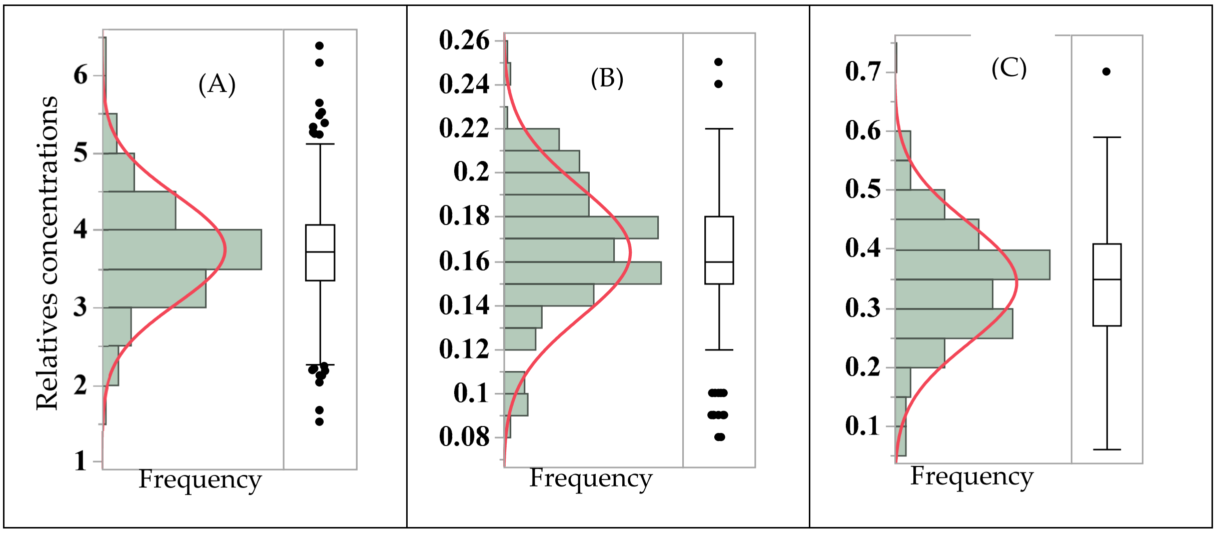

Figure S2: The box plots and the histograms of the relative concentrations 2-MeF/furan (A), 3-MeF/furan (B) and 2,5-dMeF/furan (C) in coffee products (n=276). Legend: The center line in the Box plot represents the median; box limits represent the upper and the lower quartiles; whiskers are 1.5x interquartile range; and points are outliers.

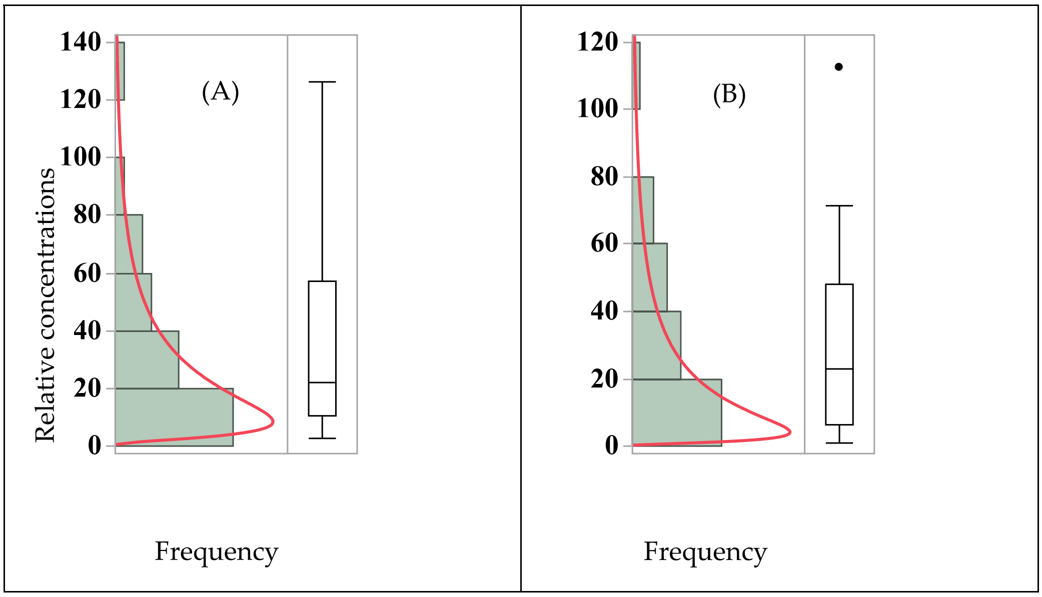

Figure S3: The box plots and the histograms of the relative concentration 2-EtF/furan (A), 2-EtF/2-MeF (B) in processed fish products (n=29). Legend: The center line in the Box plot represents the median; box limits represent the upper and the lower quartiles; whiskers are 1.5x interquartile range; and points are outliers.

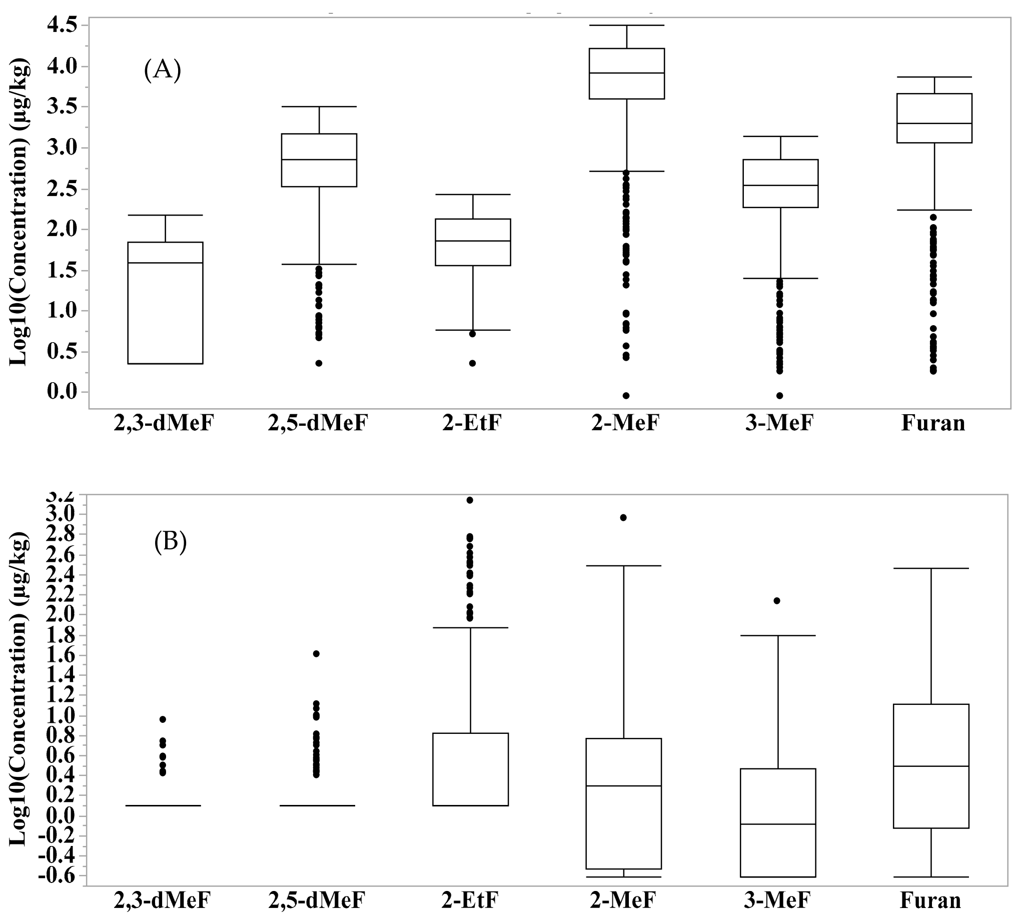

Figure S4: The Box plots of the Logarithm of the individual compound concentration in the coffee (A), and in other food groups (B).

Legend: Box plot elements, center line represents the median; box limits represent the upper and the lower quartiles; whiskers are 1.5x interquartile range; and point is an outlier.

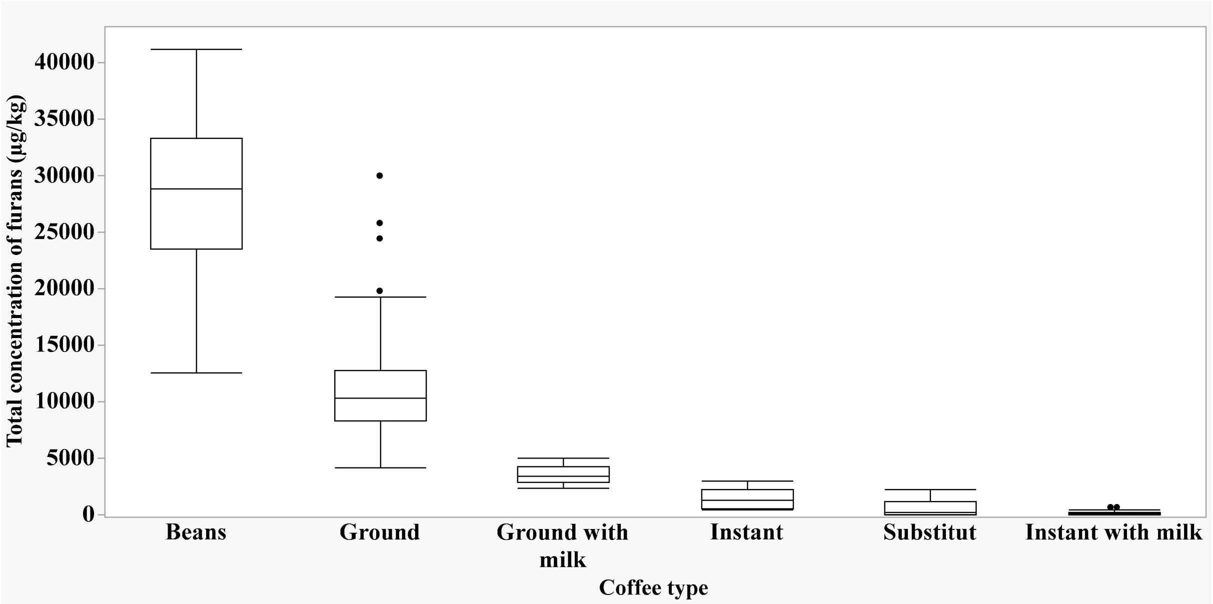

Figure S5: The box plots of the total concentrations of furan compounds in coffee products (solid) (n=280)

Legend: Box plot elements, center line represents the median; box limits represent the upper and the lower quartiles; whiskers are 1.5x interquartile range; and point is an outlier.

Table S1 The frequency of the number of furan compounds detected in coffee versus other foods

Nb. Compounds (cut-off)	Nb. occurrence in coffee	Nb. occurrence in other foods
0	0	89
1	4	87
2	13	120
3	9	139
4	27	242
5	70	20
6	180	3
Total	303	700

Table S2 The percent of the presence / absence of furan compounds (n: 1003)

	Furan	2-MeF	3-MeF	2-EtF	2,5-dMeF	2,3-dMeF
Absence	14%	18%	31%	46%	69%	81%
Presence	86%	82%	69%	54%	31%	19%

Table S3 The total concentration (μg/kg) of the selected furan compounds in the different food groups

Level	Minimum	Median	Maximum
Coffee and imitates (solid)	10.5	12255.2	41146.3
Seafood	LOQ	42.7	1522.6
Bakery	LOQ	15.5	1255.6
Coffee beverages	10.4	94.2	955.2
Composite	LOQ	17.7	419.2
Retail baby food	LOQ	16.3	197.9
Snacks	LOQ	18.6	191.1
Herbs and condiments	LOQ	19.2	174.5
Sugars and oils	LOQ	5.6	147.0
Grains	LOQ	13.4	85.8
Beverages	LOQ	5.6	17.1

Table S4 The hazard index for the selected furan compounds in the different food groups

**Food matrix**	**Number of samples**	**Min-Max**	**Median**
Coffee and coffee imitate (beverages)	303	≤0.001-0.570	0.068
Seafood	53	≤0.001-0.340	0.010
Spices and condiments	67	≤0.001-0.039	0.004
Snacks	108	≤0.001-0.043	0.004
Composite	107	≤0.001-0.094	0.004
Baby food (retails)	110	≤0.001-0.044	0.004
Bakery	92	≤0.001-0.280	0.003
Grains	83	≤0.001-0.019	0.003
Sugars and oils	14	≤0.001-0.033	0.001
Beverages	41	≤0.001-0.004	0.001
Baby food (homemade)	25	≤0.001-0.002	0.001
